# Circulating Levels of Soluble Receptor Activator of NF-**κ**B Ligand and Matrix Metalloproteinase 3 (and Their Antagonists) in Asian Indian Patients with Ankylosing Spondylitis

**DOI:** 10.1155/2013/814350

**Published:** 2013-09-01

**Authors:** Varun Dhir, Rajni Srivastava, Amita Aggarwal

**Affiliations:** ^1^Department of Clinical Immunology, Sanjay Gandhi Post Graduate Institute of Medical Sciences, Lucknow 226014, India; ^2^Department of Internal Medicine, Postgraduate Institute of Medical Education and Research, Chandigarh 160012, India

## Abstract

*Background*. Bone loss in ankylosing spondylitis may be related to inflammation. Data from previous studies on circulating levels of sRANKL, OPG, MMP3, and TIMP is inconsistent; thus this study is planned to look at this aspect in Asian Indian patients. *Methods*. Cross-sectional study included patients with ankylosing spondylitis and age- and gender-similar controls. Serum levels of sRANKL, OPG, MMP-3, and TIMP-1 were measured by ELISA. *Results*. Included 85 patients (M : F = 82 : 3) having mean age (±SD) 33.0 ± 10.0 years and disease duration 11.3 ± 7.3 years. BASDAI, BASFI, BASMI, and ESR were 4.0 ± 2.2, 3.9 ± 2.8, 3.0 ± 2.8, and 59.2 ± 31.2, respectively. Patients had higher mean (±SD) OPG level (649.7 ± 286.8, 389.3 ± 244.8 pg/mL, *P* < 0.001). However, there was no difference in sRANKL (349.2 ± 872.0, 554.7 ± 1850.1, *P* = ns). Serum MMP-3 (91.4 ± 84.7, 55.9 ± 37.1 ng/mL, *P* < 0.01) and TIMP-1 (520.6 ± 450.7, 296.5 ± 114.2 ng/mL, *P* < 0.001) levels were higher in patients; however, there was no difference in MMP-3/TIMP-1 ratio. *Conclusion*. Circulating levels of OPG were higher; however, there was no difference in sRANKL in Asian Indian ankylosing spondylitis patients. Although both MMP-3 and TIMP-1 were raised, their ratio was not different from that of controls.

## 1. Introduction

Patients with ankylosing spondylitis have been shown to have low bone mineral density at spine and propensity for vertebral fractures. The loss of bone mineral density has been shown to be more marked in late than early disease [1]. The bone loss may be related to inflammation, as in other chronic inflammatory diseases [2, 3]. Indeed, ankylosing spondylitis is characterized by chronic inflammation, as evidenced by elevated proinflammatory cytokines like tumour necrosis factor-*α* (TNF*α*) and interleukin-6 [4]. These may lead to bone loss by increased expression of receptor activator of NF-*κ*B ligand (RANKL) on osteoblasts and stromal cells and its soluble form (sRANKL). RANKL and cytokines lead to osteoclast and other inflammatory cell activation and release of bone and cartilage degrading enzymes like cathepsin K and matrix metalloproteinases (MMPs) [5, 6]. Their natural antagonists, that is, osteoprotegerin (for RANKL) and tissue inhibitor of metalloproteinase or TIMP (for MMP) oppose their actions. Among the MMPs, there is promising data on the association of disease activity with MMP-3 [7]. There is inconsistent data on circulating levels of these molecules in ankylosing spondylitis, especially in Asian Indians. Thus this study was planned to look at levels of these molecules in this population.

## 2. Methods

### 2.1. Study Design

This cross-sectional study was carried out in a North Indian university hospital between April 2010 and February 2011. Institutional ethical clearance was taken and all patients gave written consent.

### 2.2. Subjects

Consecutive patients with ankylosing spondylitis, attending the outpatient rheumatology clinic, and who gave consent were recruited. Patients fulfilled 1984 modified New York Criteria for Ankylosing Spondylitis [8]. Patients with comorbid conditions/intake of drugs known to affect bone mineral density like renal failure, use of bisphosphonates, hypo/hyperthyroidism, or inflammatory bowel disease were excluded. Patients with “bamboo spine” (radiographs of the lumbar and thoracic vertebra showing syndesmophytes at all intervertebral levels from T6 to S1) were also excluded. In addition 20-age and gender-similar controls were enrolled.

### 2.3. Clinical Assessment

Disease activity was assessed using the “Bath Ankylosing Spondylitis Disease Activity Index” (BASDAI). This consists of 6 questions on 5 major symptoms of spinal pain, joint pain, entheseal pain, fatigue, and early morning stiffness [9]. Function was assessed by the “Bath Ankylosing Spondylitis Functional Index” (BASFI), which comprises 10 questions on function and ability to cope with everyday life [10]. Answers to both BASDAI and BASFI were given on 10 cm visual analog scale. Metrology was assessed using the “Bath Ankylosing Spondylitis Metrological Index,” which comprises measurement of lumbar flexion (modified Schober's test), intermalleolar distance, cervical rotation, lumbar side flexion, and tragus-to-wall distance [11].

### 2.4. Laboratory Data

A radiograph of lumbosacral spine to detect syndesmophytes was done. Most were tested for presence of the gene HLA-B27 using polymerase chain reaction. Erythrocyte sedimentation rate (Westergren method) was noted. Serum was separated and stored at −80°C. Soluble receptor activator of NF-Kappa B (sRANKL) was determined by sandwich ELISA (Komabiotech, Republic of Korea). Osteoprotegerin was determined using sandwich ELISA (R&D, Duo set) with range 62.5–4000 pg/mL. MMP-3 and TIMP-1 were estimated by ELISA using a commercial kit (R&D Systems, Minneapolis, MN, USA). The MMP-3 kit detected both active and pro-MMP-3 (total MMP-3) with range of 31.25–2000 pg/mL.

### 2.5. Statistical Analysis

Analysis was done using SPSS version 15. Student's *t*-test was used to compare means and correlation was done by Pearsons correlation.

## 3. Results

The study included 85 patients (M : F = 82 : 3) of ankylosing spondylitis with mean age (±SD) 33.0 ± 10.0 years. Mean duration of disease was 11.3 ± 7.3 years and time since diagnosis was 3.8 ± 5.1 years. The means (±SD) of BASDAI, BASFI, BASMI, and ESR were 4.0 ± 2.2, 3.9 ± 2.8, 3.0 ± 2.8, and 59.2 ± 31.2 mm, respectively. Syndesmophytes were present in 36 (42%) patients and HLA-B27 positive in 95% (59 of 63 tested). 

Patients had higher levels of osteoprotegerin (OPG) than controls. However, sRANKL were similar in both patients and controls, being detectable in only a quarter of both (Table 1). Even on categorizing patients by BASDAI into three categories of high, moderate, and low (BASDAI ≤ 4, BASDAI 4–6, BASDAI ≥6, resp.), sRANKL was detected in an equal proportion in all three groups. Levels of both serum MMP-3 and TIMP-1 levels were higher in patients compared to controls. But, there was no difference in the MMP-3 to TIMP ratio (Table 1). There was no correlation between BASDAI and levels of OPG (*r* = −0.05), MMP-3 (*r* = −0.04), or TIMP-1 (−0.01, *P* = *ns*). There was also no correlation between ESR and OPG (*r* = −0.12) or MMP-3 (*r* = 0.09, *P* = *ns*). However, TIMP-1 correlated with ESR (*r* = 0.30, *P* = 0.009).

## 4. Discussion

This study found higher levels of osteoprotegerin (OPG) in patients of ankylosing spondylitis than controls; however, sRANKL was detected in only a minority in both. Matrix metalloproteinase 3 (MMP-3) and tissue inhibitor of metalloproteinase 1 (TIMP-1) were both elevated in patients; however, there was no difference in the MMP-3/TIMP-1 ratio. 

This study found higher levels of OPG in ankylosing spondylitis patients compared to controls. This is similar to what most of the previous studies have found [12–14]. However, some studies have found no difference or lower levels compared to controls [15, 16]. The reason for these differences may be related to disease duration, which was around 10 years in our study as well as the other studies showing raised OPG but was only 5 years in the latter study. Indeed, even on immunohistochemistry, a high expression of OPG has been shown in synovial macrophage-type synovial lining cells and endothelial cells in patients of spondyloarthropathy [17]. Another study also confirmed high levels of OPG in joints of patients with spondyloarthritis [18]. Thus, it seems the high serum levels do reflect the high levels in the joints. We did not find any correlation with disease activity, similar to most other studies [12, 18]. However, some have found a correlation with acute-phase markers [14]. Maksymowych et al. have looked at markers to predict radiographic damage progression using 2-year data scored using the modified Stoke AS Spine Score (mSASSS) [19]. They found MMP-3 but not OPG to predict damage. It would have been interesting to look at the cross-sectional association of MMP-3 and OPG with mSASSS; however, we did not obtain this data.

This study found the values of sRANKL to be similar in patients and controls. This is different from previous studies, most of which found higher levels in patients compared to controls [14, 15]. The reason may be related to the detection limit of sRANKL by the kit we used, which captured only free sRANKL which constitutes only 1/1000 of the total serum sRANKL (other being bound to proteins like OPG) [6]. On the other hand, it is possible that sRANKL is not overexpressed in ankylosing spondylitis in serum and the local tissues. Indeed, a study on spinal tissue obtained during surgery in AS patients did not find sRANKL expression in any patient sample except one [20]. Another study found disconnect between mRNA levels and serum levels in HLAB27 transgenic rats [21]. We could not assess correlation of sRANKL with disease activity measures, because it was undetectable in a majority. Previous longitudinal studies of anti-TNF agents over 3–6 months, with control of disease activity, have not found any change in levels of OPG or sRANKL [22, 23].

This study found higher levels of both MMP-3 and TIMP-1 in ankylosing spondylitis. High levels of MMP-3 have been shown in serum of ankylosing spondylitis patients compared to controls [7, 24]. Immuno-histochemistry in peripheral synovitis of spondarthritis patients also shows high levels of MMP-3, with downregulation after biological treatment [24]. MMP-3 leads to extracellular matrix degradation and activates other pro-MMPs [25]. It has been shown that apart from Cathepsin K, MMPs have a role in bone matrix degradation leading to bone loss [26]. Indeed, immunohistochemical studies on spinal tissue from AS patients do show increased mononuclear cells expressing matrix metalloproteinase 1 and 3 [20]. We did not find any correlation with measures of disease activity. Among previous studies, there has been inconsistent association of MMP-3 with disease activity parameters. Some studies found a correlation of MMP-3 with both BASDAI and acute-phase reactants [7], some with only acute-phase reactants not with disease activity [27], some with BASDAI but not acute-phase reactants [28] and some with none [29]. 

To conclude, this study did not find elevated circulating levels of sRANKL or elevation in the MMP-3/TIMP ratio in ankylosing spondylitis patients. However, a study of bone density and radiological damage with these markers may provide a better understanding of the role of these markers in bone loss in ankylosing spondylitis.

## Figures and Tables

**Table 1:** Serum level of soluble receptor activator of NF-*κ*B ligand (sRANKL), osteoprotegerin (OPG), matrix metalloproteinase 3 (MMP-3), and tissue inhibitor of metalloproteinase 1 (TIMP-1) in patients of ankylosing spondylitis versus controls.

	Ankylosing spondylitis (*n* = 85)	Controls (*n* = 20)	*P *
OPG pg/ml mean ± SD	649.7 ± 286.8	389.3 ± 244.8	<0.001
sRANKL pg/ml mean ± SD^a^	349.2 ± 872.0	554.7 ± 1850.1	0.50
MMP-3 ng/ml mean ± SD	91.4 ± 84.7	55.9 ± 37.1	0.006
TIMP-1 ng/ml mean ± SD	520.6 ± 450.7	296.5 ± 114.2	0.001

^a^sRANKL was above the lowest detectable limit in only one-fourth of the samples (done in 55 patients and 20 controls). In others the lowest detectable limit by kit (32.25 pg/ml) was taken to represent the value of the sample.
